# Animal Pesticide Poisoning in Tunisia

**DOI:** 10.3389/fvets.2019.00369

**Published:** 2019-11-05

**Authors:** Rym Lahmar, Philippe Berny, Tarek Mahjoub, Samir Ben Youssef

**Affiliations:** ^1^Pharmacy-Toxicology, University of Manouba, National School of Veterinary Medicine of Sidi Thabet, Sidi Thabet, Tunisia; ^2^Pharmacy-Toxicology, Université de Lyon, VetAgro Sup, Lyon, France; ^3^Biochemistry, University of Manouba, National School of Veterinary Medicine of Sidi Thabet, Sidi Thabet, Tunisia

**Keywords:** intoxication, pesticides, domestic animals, livestock, Tunisia

## Abstract

During the period from 2014 to 2017, a retrospective study on pesticide poisoning in domestic animals and livestock was compiled and then analyzed. A total of 71 pesticide analyses have been submitted to the Pharmacy and Toxicology Laboratory of the School of Veterinary Medicine of Sidi Thabet in Tunisia. All the cases were first referred either through the clinical and/or pathological departments of the Veterinary School, the private and/or governmental veterinarians or directly by the pet owners. Among the total number of the suspected samples, 21 (29.6%) cases were found positive for various kinds of pesticides. Carbamate insecticides were the most frequently implicated pesticide (52.4% of the total positive cases), followed by organophosphate insecticides (19%), then rodenticides-anticoagulants and rodenticides non-anticoagulants (14.3% each). Therefore, carbamates and organophosphates are the most implicated group of pesticides in intoxications (71.4%). Among the 21 positive cases were 11 dogs, 4 cats, 3 poultry, 2 ruminants, and 1 case of bee poisoning. Partition chromatography (HPLC) has been used to characterize the incriminated pesticides. The aim of this survey was to determine incidence and characteristics of pesticide poisoning in domestic and farm animals in Tunisia. The reported results are useful for epidemiological cartography and medical management of intoxicated animals.

## Introduction

Pesticides are widely used to control harmful pests in agriculture and in both the professional and domestic environment. In most cases, misuse or accidental exposure are the common causes of pesticide poisoning (1).

Several retrospective studies about animal poisoning have been conducted around the world, in Europe (2–6), Australia (1, 7), USA (8), Brazil (9), and Canada (10), but there are no data from the North African countries, including Tunisia.

Poisoning by pesticides in humans has been frequently reported in Tunisia. Acute pesticide poisoning occupies second place after those due to drugs, representing 13.3% of total chemical poisonings (11). However, there is no information about intoxications in domestic animals and livestock.

Thus, our paper represents the first report on original data in this geographical area.

In Tunisia, animal poisoning is diagnosed only in the Pharmacy and Toxicology Laboratory at the National School of Veterinary Medicine. There are no other laboratories in all the country where pesticides in animals are analyzed. When intoxication cases are suspected by a veterinarian, relevant samples, such as liver, gastric content or baits, are referred to the Veterinary School for confirmation. The majority of pesticides are identified by High Performance Liquid Chromatography (HPLC) techniques using commercially available standards and using colorimetric reactions for some older toxic compounds.

All clinical cases are registered in the laboratory and then stored in the archives.

The purpose of this study was to report the incidence and characteristics of veterinary intoxications in Tunisia reported by the Laboratory of Pharmacy and Toxicology at the National School of Veterinary Medicine, from January 1st 2014 to December 31st 2017.

## Materials and Methods

Seventy-one cases of suspected intoxications, received during 4 years from January 2014 to December 2017, were analyzed.

**Figure 1:**
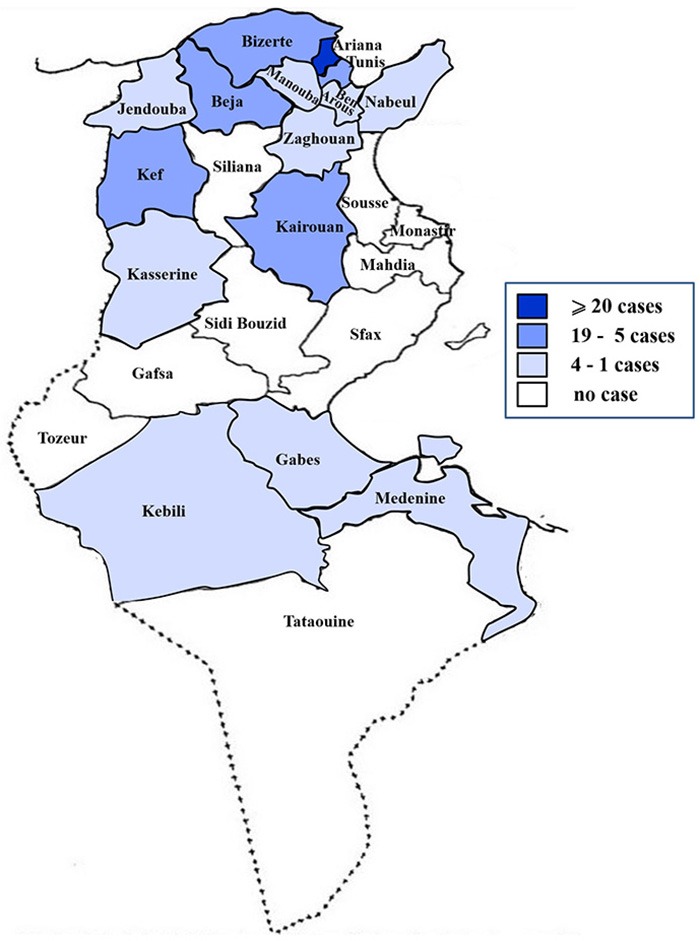
Map of Tunisia showing origin of analyzed samples during 2014–2017.

Samples originated from the whole country, mainly Ariana governorate (29%), then Bizerte (12 %), Tunis (10 %), Beja and Kairouan (9% each) governorates (Figure 1).

For each case we received, there was one or many types of biological samples such as liver, gastric content, bait (meat, fish and bread), vomitus, urine, and bees.

Dogs, cats, poultry, cattle, sheep, and bees were among the intoxicated animals.

Necropsy was performed on animals in <24 h, either on the field by veterinarians or in the anatomic pathology laboratory at the National School of Veterinary Medicine. The veterinary pathologist reported description, in the case of suspected substances intermingling with gastric content (abnormal color, suspicious granules), or suggestive lesions of certain intoxications (hemorrhagic lesions, acute pulmonary edema with tracheal foam). Necropsy findings are not specific in animal poisoning, but they may lead to diagnosis of the poisoning (12). The main findings of the necropsies are exceptionally photographed, then all autopsy reports are classified in the anatomic pathology laboratory archives.

Data provided by the owners are very important for the poisoning's diagnosis (history of pesticide use, suspect bait found near the animal, description of symptoms, etc.).

Usually, the presence of unknown bait intentionally placed near the animal leads to suspicions of criminal intoxication.

The integration of epidemiological, clinical, and necropsy factors allows us to reduce the toxic substances potentially involved in poisoning.

In this context, the detection of a foreign substance in the body confirms intoxication. The analysis was conducted with the available chromatographic techniques at the National School of Veterinary Medicine of Sidi Thabet in Tunisia. The analyzed samples were extracted from tissue (liver), solid (bait), or liquid samples (gastric content, vomitus, urine). The actual proof of presence or absence for a suspected pesticide was performed by liquid-liquid extraction with ethyl ether (13) followed by separation and characterization by HPLC (14) with UV detection (Agilent technologies 1200 series). This is achieved using a photodiode array detector (15) to obtain a full-UV spectrum for identification.

According to the type of sample, the mean recovery of a method was in the range of 50–60%. Limits of detection (LOD) were 0.04–0.08 mg/kg for HPLC analyses and >5 mg/kg for colorimetric reactions (Table 1).

**Table 1:** Information's lists on applied determination techniques for different samples.

**Pesticide class**	**Analyte**	**Type of sample**	**Final determination technique**	**Analyte recovery (%)**	**Limit of detection LOD (mg/Kg)**
Carbamate insecticides	Carbaryl Carbofuran Methomyl	Liver gastric content	HPLC -UV	>50	>0.04
Organophosphate insecticides	Dichlorvos malathion Diazinon	Liver gastric content	HPLC -UV	>50	>0.04
Rodenticides-anticoagulants	Chlorophacinone Bromadiolone Brodifacoum	Liver gastric content	HPLC -UV	>60	0.02–0.08
Rodenticides non-anticoagulants	Chloralose	Urine	Colorimetric reaction: Fujiwara-Ross (16)	>95	>5
Molluscicides	Metaldehyde	Bait gastric content	Colorimetric reaction with sulfuric acid and guaiacol (17)	>50	>10

Toxicological investigations were carried out in our laboratory using qualitative and non-quantitative methods. The only detection of a foreign substance to the body is sufficient to confirm suspicion of intoxication; thus, limits of quantification (LOQ) are not of a great importance in our case.

This laboratory has a narrow range of standards, which are frequently used in Tunisia. We searched for carbamates (Carbaryl, Carbofuran, Methiocarb, Methomyl, and Pirimicarb), organophosphates (Chlorpyriphos, Dichlorvos, Diazinon, Dimethoate, Malathion, and Parathion), anticoagulant rodenticides (Chlorophacinone, Brodifacoum, Bromadiolone, Difenacoum, and Warfarin), and other insecticides (Deltamethrin).

Chloralose, Metaldehyde, and Strychnine analyses were performed only by colorimetric reactions.

All analyzed cases were recorded in the archives of the toxicology laboratory. All data used in the present study were reviewed and managed using Microsoft Access 2016.

## Results

### Descriptive Study of Recorded Cases

Among the 71 received cases, 62 (87.3%) were biological samples from suspected animals' poisoning (related or not with bait), and 9 (12.7%) were just suspected baits. Analysis revealed 21 (29.6%) positive cases containing pesticides. Confirmed animal poisoning cases reached 18/62 (29%), while the presence of a toxic agent was detected in 3/9 baits (33.3%).

**Figure 2:**
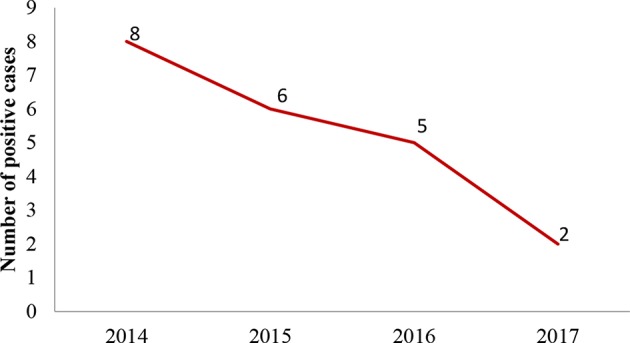
Yearly distribution of confirmed animal poisoning from 2014 to 2017.

**Figure 3:**
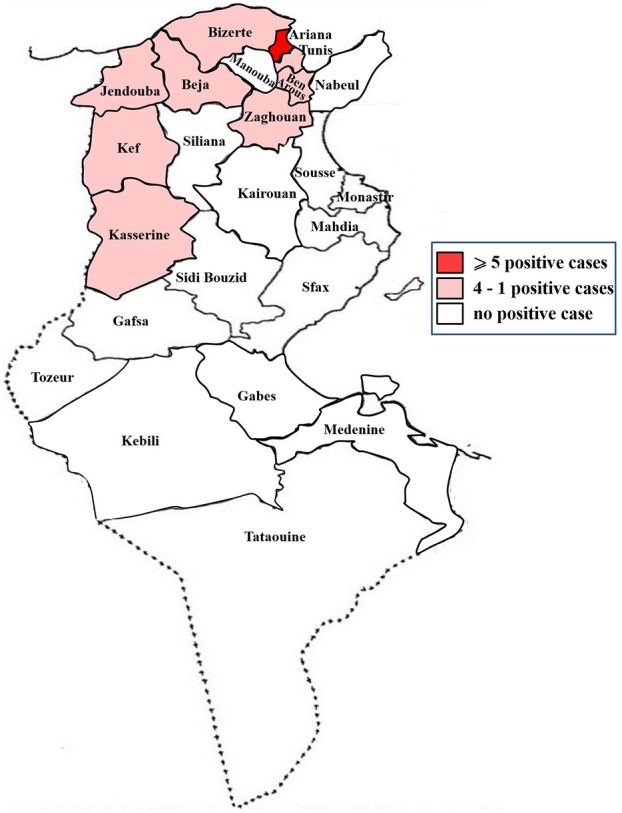
Map of Tunisia showing origin of positive samples analyzed from 2014 to 2017.

Confirmed positive cases submitted to the laboratory were eight in 2014, six in 2015, five in 2016, and two in 2017 (Figure 2). They originated from the north and the western-central part of Tunisia (Figure 3).

### Classification of Samples

From January 2014 to December 2017, a total of 71 cases of suspected intoxications was analyzed. For each case, we received one or many types of samples: 51 liver samples, 49 gastric contents, 16 baits (meat, fish and bread), 3 vomitus, 1 urine and bees. Thus, 121 analyses were performed; 39 of them were positive, which confirmed the 21 cases of poisoning. Among the 121 analyzed samples, 42% corresponded to livers, 41% to gastric contents, 13% to baits, and 4% to other samples like urine and carcasses.

Among 14 positive cases with multiple samples, 11 had positive results for all samples (stomach contents, liver, and/or baits) and 3 had positive results only for stomach contents and baits but nothing in the liver.

Among the 16 baits that were analyzed, 7 contained pesticides. These positive baits caused death in 4 clinical cases.

### Analysis Requests

**Figure 4:**
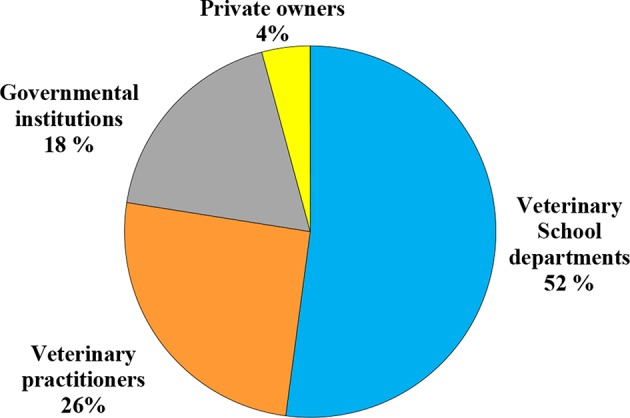
Origin of pesticide analysis requests during 2014–2017.

The requested analyses were addressed from pathology and poultry laboratories of the Veterinary School of Sidi Thabet (52%), private veterinary practitioners (26%), governmental institutions (18%) such as police offices, medical clinics and hospitals, regional governorates for agricultural development, state land offices, public health authorities, and private owners (4%) (Figure 4).

### Circumstances of Animal Poisoning

**Figure 5:**
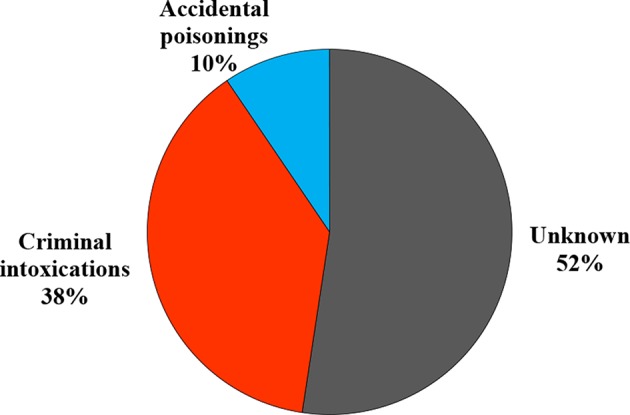
Circumstances of animal poisoning.

Confirmed poisoning cases were due to unknown circumstances (52%), criminal intoxications (38%) and accidental exposures (10%) (Figure 5).

Accidental poisoning was even due to the misuse of pesticides (use of rodenticides, insecticides in agriculture or pesticides that were stored improperly).

On the other side, the presence of an unknown suspect bait placed intentionally near the animal suggests criminal intoxication.

### Animal Species Involved

In total, 71 animals were submitted for investigation, and 21 animals were found positive for pesticide intoxication, including 11/35 dogs, 4/5 cats, 3/11 poultry, 2/13 ruminants, and 1/7 bees among other species (3 horses, 2 boar and land tortoise). Thus, 71% of confirmed cases were pets and 29% were farm animals.

The case of the bees is a special case. In fact, following the spreading of insecticides (Carbofuran) to treat fruit trees in Ben Arous governorate in the northern-east of the country, there was a significant mortality of bees living nearby, and in order to benefit from the insurance, the breeder must prove this accidental cause of the bees' death.

### Seasonal Trend

**Figure 6:**
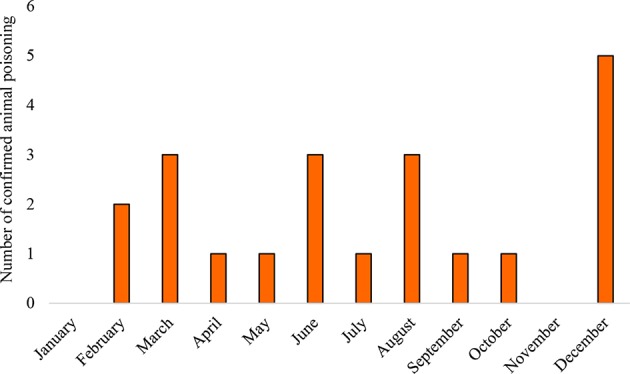
Mean number of confirmed animal poisoning cases per month during 2014–2017.

The peak of confirmed intoxications during the period (2014–2017) occurred in December (Figure 6).

### Toxic Agents Involved

This work reported that the main family of pesticide incriminated in animals' poisoning was carbamate insecticides (52.4%), with methomyl in the lead with 33.3% of positive pesticide tests (Table 2).

**Table 2:** Pesticides implicated in animal poisoning and poisoned baits from 2014 to 2017.

**Toxic agents**	**Number of detected cases**	**Total of chemical classes (%)**
**Carbamates**		52.4
Carbaryl	1	4.8
Carbofuran	3	14.3
Methiocarb	0	0
Methomyl	**7**	33.3
Pirimicarb	0	0
**Organophosphates**		19
Chlorpyriphos	0	0
Dichlorvos	2	9.5
Diazinon	2	9.5
Dimethoate	0	0
Malathion	0	0
Parathion	0	0
**Rodenticides-anticoagulants**		14.3
Warfarin	0	0
Chlorophacinone	2	9.5
Brodifacoum	0	0
Bromadiolone	1	4.8
Difenacoum	0	0
**Rodenticides non-anticoagulants**		14.3
Chloralose	3	14.3
**Molluscicides**		
Metaldehyde	0	0
**Pyrethroids**		
Deltamethrin	0	0
**Total positive cases**	21	100

These compounds were followed by organophosphate insecticides (19%), then anticoagulant and non-anticoagulant rodenticides (each with 14.3% of the positive results).

Pesticides implicated in intoxications and found in baits are presented in Table 2.

### Clinical Signs and Lesions After Necropsy

Clinical signs and post-mortem lesions caused by the most common toxic compounds involved in animal poisonings are reported in Table 3. They are reported only for the most common toxic agents.

**Table 3:** Clinical signs and necropsy of the most common toxicants involved in animal poisoning from 2014 to 2017.

**Toxicant**	**Clinical signs (%)**	**Necropsy (%)**
Methomyl (dog)	Sudden death (40%), hyper-salivation (40%), convulsion (30%)	Hemorrhagic gastroenteritis with congestion (80%), degeneration of the liver (60%), pulmonary edema (40%)
Carbofuran	Sudden death (50%), vomiting (25%), convulsion (25%)	Hemorrhagic gastro-enteritis with congestion (80%)
Chloralose	Hypothermia (100%), coma (80%), muscle tremors (60%)	Congestion of the carcass (80%)
Dichlorvos	Sudden death	Congestion of the carcass, hemorrhagic gastroenteritis with congestion
Diazinon	Sudden death	Congestion of the carcass
Bromadiolone	Pallor, incoordination, lethargy	Anemia, hemothorax, hemopericardium
Chlorophacinone	Pallor, lethargy	Anemia, hemothorax

**Photo 1:**
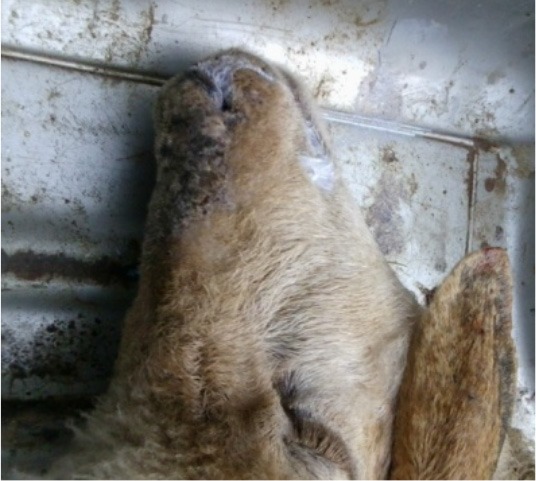
Presence of foams in the mouth and nostrils of a sheep intoxicated with methomyl.

**Photo 2:**
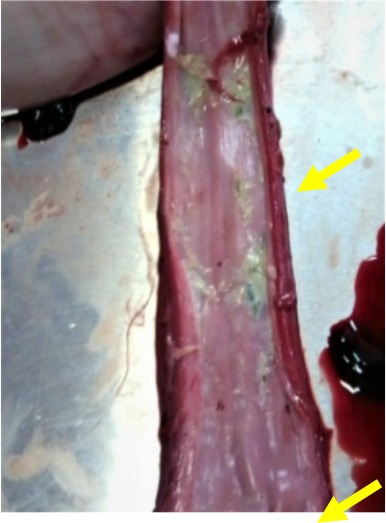
Presence of methomyl granules in the esophagus of an intoxicated sheep.

**Photo 3:**
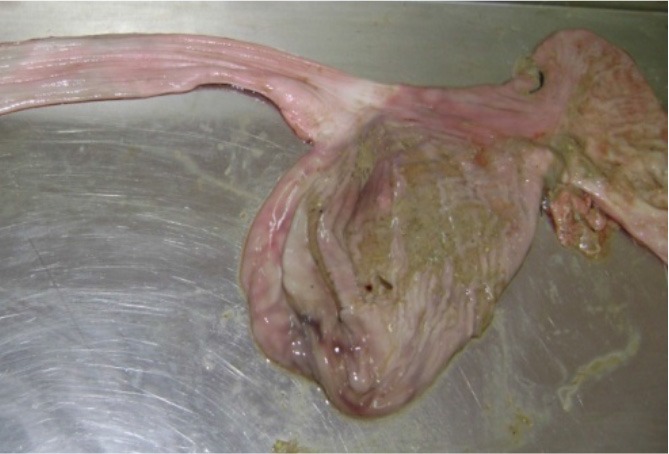
Hemorrhagic gastroenteritis of a dog intoxicated with carbamates.

**Photo 4:**
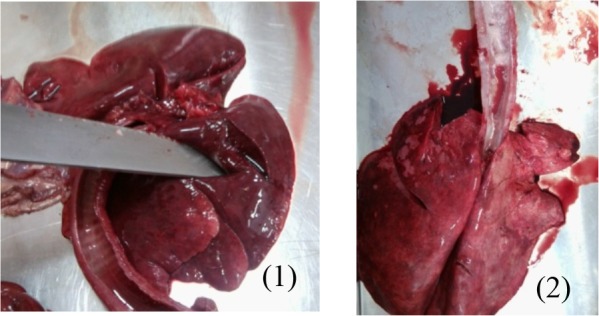
Lung edema of a dog **(1)** and sheep **(2)** intoxicated with methomyl.

**Photo 5:**
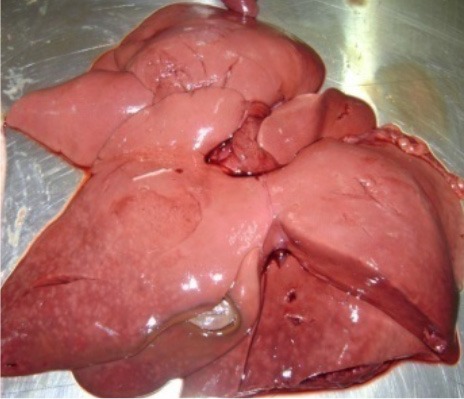
Degeneration of the dog's liver intoxicated with methomyl.

**Photo 6:**
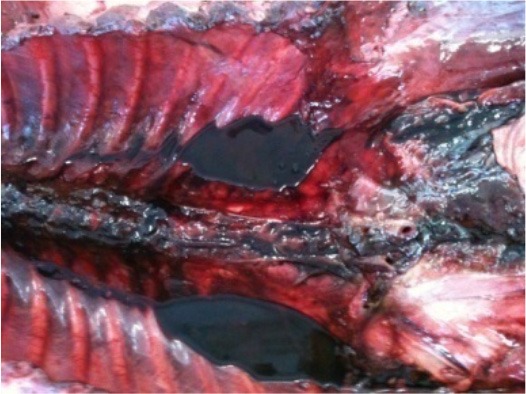
Hemothorax in a dog's liver intoxicated with chlorophacinone.

Photos 1–6 illustrate the main lesions observed during pesticide's poisoning.

## Discussion

This work presents data from recorded cases of animal pesticide poisoning and poisoned baits in Tunisia over a period of 4 years. To our knowledge, this is the first study carried out in Tunisia and in North Africa.

Among the total number of suspected samples for pesticides, 21 (29.6%) cases were found positive to contain pesticides.

The number of events registered in our study compared to those detected in other countries could be misleading. In our case, the investigated toxic agents were a limited number of pesticides, which may be due to absence of diagnostic screening for pesticides which have not been considered in our analysis. Another constraint may be the qualitative methods used (metaldehyde, chloralose), which do not have a low detection limit (LOD > 5 mg/kg).

The number of cases submitted to our laboratory seems to be decreasing from 2014 to 2017. This could be related to the improvement of the security in the country against criminal intoxications, to changes in pesticide's use or to a restriction of pesticides available in pharmaceutical and agricultural offices.

Clinical cases originated from all of Tunisia but mostly from the northern regions. This could be explained by the location of the Veterinary School, in Ariana governorate, which easily accessible, hence the highest number of cases.

Many kinds of samples were received, especially liver, gastric content and bait from our Veterinary School, mainly from the pathology department.

Liver, gastric content and bait are the best choices for samples for pesticide research, including cholinesterase inhibitors (18) and even anticoagulant rodenticides.

Contrary to what Roch (12) reported, the stomach content can be very useful for anticoagulant research. In fact, analyses of all samples of the stomach content confirmed poisoning with anticoagulants. Some baits in pasta or block presentation can remain in the stomach for a long time.

Analyses performed on multiple samples (liver, gastric contain, and/or bait) from the same positive case have all presented positive results for nearly 80% of cases, while the rest had positive results only for stomach contents and baits. Absence of compounds in the liver could be explained by the evolution's stage of the intoxication: when the poisoning is the over-acute type, a large quantity of pesticides have not yet been absorbed through the gastrointestinal mucosa, so the toxic does not have time to be well-accumulated in the liver and thus it is found mainly in the stomach.

In total, 71 animals were suspected and 21 were found positive for pesticide intoxication, including 11 dogs, 4 cats, 3 poultry, 2 ruminants, and 1 bee case. Thus, 71% of the confirmed cases were companion animals while 29% were livestock.

An analogous scenario was reported in European countries (2), such as France (19), Spain (20), Austria (1), Italy (5), and the United States of America (21). Unlike cats, dogs are not very demanding about their eating habits. In fact, this non-selective food behavior predisposes them to intoxication by poisoned baits. This trend is confirmed in this study (21).

The low number of poisoning cases observed in livestock (29%) is probably due to the fact that this animal category is not exposed to malicious intoxication, unlike dogs considered as guard animals. Furthermore, farm animals generally evolve in a well-controlled environment and the likelihood of exposure is very low (22).

Surprisingly, we received only 3 suspected samples from wildlife (2 boar and 1 land tortoise) and no positive case was reported. However, in some European countries like France or the United Kingdom, there is evidence that pesticide poisoning is a common issue in terrestrial vertebrates (23), but in these countries specialized networks exist to report pesticide poisoning and legal actions may be taken for criminal baits (24).

This work highlights the fact that carbamates are incriminated in most of the pesticide poisoning in animals, with 52.4%. Methomyl, belonging to carbamates, is the most frequently used, with 33.3% of the positive pesticide analyses. It is used indoors in a granular formulation against insect pests (2).

These compounds were followed by organophosphate insecticides at 19%, then by anticoagulant rodenticides and non-anticoagulant rodenticides with 14.3% each.

This trend of poisoning with cholinesterase inhibitors (71.4%) was reported in other countries (1, 2, 5).

This observation could be correlated with the availability of these insecticides and their current use near homes. Several formulations are on sale in supermarkets (25).

In Tunisia, pesticides used in agriculture contain about 215 approved active substances, among them 64 insecticides (26). It would also be interesting to relate the number of poisoning cases to actual sales of each pesticide.

Cholinesterase inhibitors lengthen the action of acetylcholine in the neuromuscular synaptic junction. This results in a muscarinic effect: stimulation of secretion [hypersalivation (Photo 1), diarrhea and lung edema (Photo 4)], miosis, bronchospasm, and bradycardia, a nicotine effect (convulsion, ataxia, weakness, and paralysis), and central nervous system depression with seizures (27–29). In our study, during the short clinical phase of cholinesterase inhibitors intoxication, we observed similar signs, especially salivation, convulsion, and gastro-enteritis.

Hemorrhagic lesions observed with anticoagulant pesticides (anemia, hemothorax, hemopericardium) are due to their mechanism of action: they inhibit the recycling of vitamin K1 necessary for the activation of several clotting factors. As a consequence, coagulation is impaired, hence the observed lesions (12, 30). However, contrary to Roch's (12) description, we observed in the 3 cases of anticoagulants' poisoning the presence of a friable blood clot.

Generalized congestion of the carcass and hemorrhagic gastroenteritis are often observed during oral pesticide intoxication, probably due to the caustic effect of these compounds. These 2 lesions have been described during intoxication with chloralose (31) and cholinesterase inhibitors (29).

At least 38% of confirmed poisoning cases were due to criminal intoxication.

This relatively high percentage may be related to the fact that there is only one Veterinary School in Tunisia, which contains the only laboratory of toxicology recognized by the local authorities. In addition, we could also consider the fact that only severe cases may be submitted for confirmatory analysis that are usually criminal. This percentage is quite comparable to situations described in France or the UK (24).

In urban settings, interpersonal conflicts such as neighborhood problems and intolerance toward stray or owned animals are among the main causes, whereas in rural areas poison can be used to kill animals believed to negatively affect human activities such as hunting, farming, agriculture, and truffle search (5).

Toxicological test results are official; indeed, they are most often used in legal proceedings (1).

Poisoned baits are considered a risk for public health. They constitute a danger for both target and non-target species, including humans (5).

Restrictions on many pesticides in the European Union (32, 33) may have reduced the incidence of pesticide poisoning of domestic animals (34). In a safe and eco-friendly approach, several insecticides have been banned—such as aldicarb in 2003 (32) and carbofuran in 2007 (35, 36).

Aldicarb and carbofuran, which have been prohibited by the EU, are still often reported in animal intoxications involving domestic animals (34) and wildlife (36) from Europe.

In Tunisia, the use of pesticides is controlled. The list of approved substances is defined according to the Commission's decision of April 29, 2015 (26).

Almost all the poisoning cases described in this work were fatal. The nature of the toxic substance is decisive in the prognosis. Thus, exposure to highly toxic substances, such as carbamates, is much more dangerous than anticoagulant rodenticides that could be successfully treated when they are diagnosed early (5).

In order to reduce the incidence of pesticide poisoning, it is necessary not only to ban very toxic substances but also to monitor the sale and distribution of these products. This includes import control. Additionally, it is important to decrease the active ingredient concentration in formulations of pesticides to minimize the risk of poisoning in non-target animals (37).

## Conclusion

This survey provided, for the first time, updated and useful epidemiological data on animal exposure to pesticides and in determining poisoning trends in Tunisia. A better understanding of the causes of poisoning in pets and farm animals would allow veterinary practitioners early management and establish an appropriate prophylactic strategy to hinder the advent of new cases.

The outcome of the present work showed that intentional animal poisoning with pesticides is a widespread and relevant issue in Tunisia. Carbamates still represent the main cause of animal poisoning. So, introduction of restrictive legislation in Tunisia may decrease pesticide poisoning.

## Data Availability Statement

The raw data supporting the conclusions of this manuscript will be made available by the authors, without undue reservation, to any qualified researcher.

## Ethics Statement

This study did not involve live animals since diagnostic testing was always performed on dead animals on behalf of their owners. All data were used anonymously. As such, the study did not require ethical approval.

## Author Contributions

RL: manuscript preparation and writing. PB: supervision, manuscript correction, and submission. TM: data extraction. SB: head of pharmacology/toxicology unit, Tunisia, validation of results.

### Conflict of Interest

The authors declare that the research was conducted in the absence of any commercial or financial relationships that could be construed as a potential conflict of interest.
